# Supplementary material for: Identification of photoperiod-induced specific miRNAs in the adrenal glands of Sunite sheep (Ovis aries)
Source: Front Vet Sci. 2022 Jul 22;9:888207. doi: 10.3389/fvets.2022.888207 (PMC9354845; doi:10.3389/fvets.2022.888207)
Supplement: Supplementary file 1 [file Data_Sheet_1.ZIP › Supplementary file/miRDeep2 file.zip!/miRDeep2 file/Novel_Structure/SPLP42A3/Novel_238.pdf]

Provisional ID	:	NC_019460.2_7893
Score total	:	1.7
Score for star read(s)	:	-1.3
Score for read counts	:	0
Score for mfe	:	1.4
Score for randfold	:	1.6
Score for cons. seed	:	
Total read count	:	72
Mature read count	:	72
Loop read count	:	0
Star read count	:	0

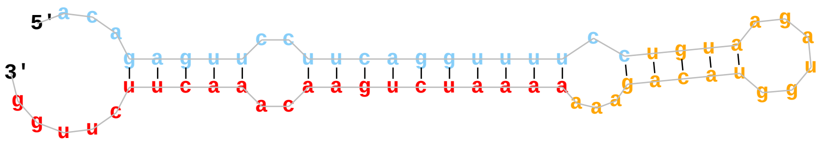

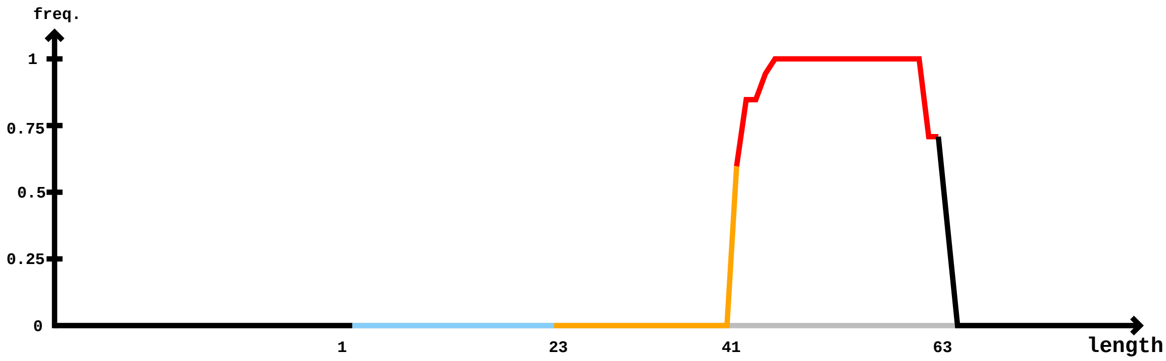

Star Mature

```
5'- gguuaguauuaugaugcacuggguuguccacagaguuccuucagguuuuccuguaagaugguacagaaaaaaaucugaacaaacuucuuggucaacccaauauuaucuguu   -3'    exp
    ..........((((((...(((((((.(((..(((((..((((((((((.(((((......)))))...))))))))))..)))))..))).))))))).)))))).....    reads    mm    sample
    .....................................................................aaaaucugaacaaacuuUuu......................    18       1     seq
    .....................................................................aaaaucugaacaaacuuUuugg....................    19       1     seq
    .....................................................................aaaaucugaacaaacuuUuuggu...................    6        1     seq
    ......................................................................aaaucugaacaaacuuUuu......................    3        1     seq
    ......................................................................aaaucugaacaaacuuUuugg....................    14       1     seq
    ......................................................................aaaucugaacaaacuucuuggu...................    1        0     seq
    ........................................................................aucugaacaaacuuUuuggu...................    7        1     seq
    .........................................................................ucugaacaaacuuUuuggu...................    4        1     seq
```
